# Landscape Movements of Migratory Birds and Bats Reveal an Expanded Scale of Stopover

**DOI:** 10.1371/journal.pone.0027054

**Published:** 2011-11-03

**Authors:** Philip D. Taylor, Stuart A. Mackenzie, Bethany G. Thurber, Anna M. Calvert, Alex M. Mills, Liam P. McGuire, Christopher G. Guglielmo

**Affiliations:** 1 Acadia University, Wolfville, Canada; 2 Bird Studies Canada, Port Rowan, Canada; 3 University of Western Ontario, London, Canada; University of Jyväskylä, Finland

## Abstract

Many species of birds and bats undertake seasonal migrations between breeding and over-wintering sites. En-route, migrants alternate periods of flight with time spent at stopover – the time and space where individuals rest and refuel for subsequent flights. We assessed the spatial scale of movements made by migrants during stopover by using an array of automated telemetry receivers with multiple antennae to track the daily location of individuals over a geographic area ∼20×40 km. We tracked the movements of 322 individuals of seven migratory vertebrate species (5 passerines, 1 owl and 1 bat) during spring and fall migratory stopover on and adjacent to a large lake peninsula. Our results show that many individuals leaving their capture site relocate within the same landscape at some point during stopover, moving as much as 30 km distant from their site of initial capture. We show that many apparent nocturnal departures from stopover sites are not a resumption of migration in the strictest sense, but are instead relocations that represent continued stopover at a broader spatial scale.

## Introduction

Billions of birds and bats undertake seasonal, nocturnal migrations between over-wintering and breeding grounds. During migration, periods of flight alternate with time spent at ‘stopover’ – the time and space where individuals rest and refuel for a subsequent migratory flight. Because time and energy costs are elevated during stopover relative to flight [1], most migration mortality probably occurs as a result of decisions made during that period [2]. As a consequence, the importance of stopover for the ecology and conservation of migrants is an area of active research [3], [4].

A ‘stopover bout’ can be thought of as the time spent between migratory flights [5]. On a fine scale, the site where an individual is found at any particular time during a stopover bout can be thought of as its ‘stopover site’. More broadly, the space occupied during a stopover bout can be considered its ‘stopover landscape’. A stopover landscape can be spatially and temporally equal to or larger than a stopover site [6]. The difficulty with these definitions is in determining whether an individual has undertaken a true migratory flight as opposed to a flight that only involves changing stopover sites within the stopover landscape.

For some species, notably waders and ducks, individuals may stop over at well-defined geographical locations where it may be more reasonable to assume that a flight away from the site represents a continuation of migration (e.g. Western Sandpiper [7], but see [8]). However, for many other species (such as most passerines and bats), the initiation of a migratory flight is more difficult to determine so the spatial and temporal scales of stopover are not as well defined. Because of this difficulty, most researchers appear to assume that when an individual leaves a stopover site, it is continuing migration. While this assumption may hold at islands or habitat patches with clear boundaries [9]–[11], in other studies, at sites where individuals have been observed to move beyond small monitored areas prior to initiating a migratory flight [12]–[15] this assumption appears to be violated.

Permanent local emigration out of a monitored study site has been recognized as a confounding factor in studies of stopover, but the frequency and spatial extent of such relocations within the stopover landscape, and the mechanisms involved, are not well known [10], [15], [16]. Some relocations may occur via small-scale diurnal foraging and exploratory movements [14], [17], while others occur via relocation flights [6], [18]. At least some of these ‘relocation flights’ are likely ‘reverse migrations’, where individuals are thought to change migration direction upon encountering adverse weather conditions or ecological barriers [19]–[22]. While such movements may not represent continuation of migration, they are thought to occur shortly (<24 h) after the end of a migratory flight, implying that these individuals have not yet settled at a particular stopover site [10], [15], [16], [23]. Although it has been recommended that inference be drawn only from individuals whose arrival and departure timing is known [24], identifying arrival and departure states with certainty is difficult.

In previous work [6] we showed that some individuals of two passerine species, Swainson's Thrush (*Catharus ustulatus)* and Hermit Thrush (*C. guttatus)* undertake nocturnal flights that result in landscape-scale relocations of up to 30 km from the site of initial capture. We have subsequently conducted a series of follow-up studies at different seasons, using different taxa, designed in part to clarify the frequency, true spatial extent and taxonomic breadth of these landscape-scale relocations. Using an automated telemetry system, we monitored the daily location of seven species of small- to medium-sized, nocturnal vertebrate migrants at a broader spatial scale than is typically employed (∼20×40 km). The continuous and simultaneous use of multiple antennae on multiple towers allowed us to distinguish probable migratory departure flights from flights where individuals simply changed stopover sites within the stopover landscape. We studied the movements of radio-tagged individuals of five passerines, one owl, and one bat, some during both spring and fall migration. These provide a comprehensive set of data with which to view the spatial and temporal scale of migratory stopover within a diverse group of taxa, during stopover bouts that have been more comprehensively observed than in most previous studies.

## Materials and Methods

### Study site and the digital array

**Figure 1:**
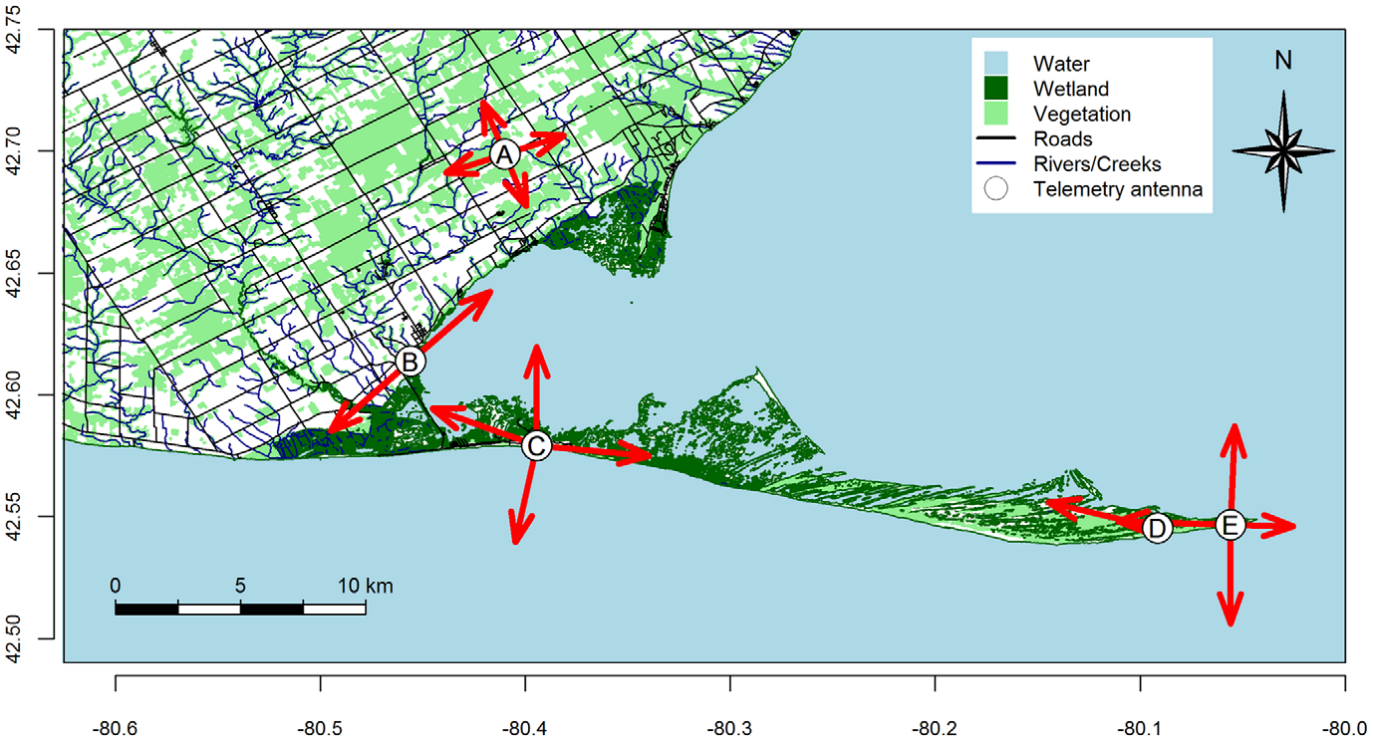
Long Point, Lake Erie, ON, Canada showing tower locations (Points A–E) used in fall 2009. The directions each antenna were pointing (fall 2009) are shown with red arrows. In fall 2008 and spring 2009 towers A and B were not present and tower D was fitted with 4 antennae and situated approximately 4 km W of the location noted. Banding locations were within 200 m of towers as follows: Mainland (A), Old Cut (C), Tip (E).

The study was conducted in and around Long Point, Ontario, Canada (42°34′ N, 80°13′ W). Long Point is a largely uninhabited sand peninsula that extends ∼40 km into Lake Erie from the north shore of the lake. The surrounding mainland comprises a mixture of deciduous forest and agricultural land (see Figure 1). Three towers (∼12 m) were erected on the point proper for fall (2008) and spring (2009). Two additional towers were erected on the adjacent mainland during fall 2009. All terrestrial locations on the point itself were within ∼12 km of a tower. Each tower contained 1–4 directional Yagi antennae (5 and/or 9 element) and was equipped with an SRX 600 automated receiver and an 8-port switchbox (ASP-8) for sequentially monitoring multiple antennae (Lotek Wireless, Newmarket, Ontario). One tower in fall 2009 had an SRX DL receiver and a single antenna. Receivers were programmed to cycle through antennae at regular intervals, just slightly greater than the burst rate employed in the study. So, for example, on a receiver with four antennae, when we were using a nominal burst rate of 5 s, the receiver would ‘listen’ to each antenna for a duration of 5.5 s every 22 s.

Daily locations were also manually obtained for each tagged bird within the range of the digital array. Daily roost locations for bats were only obtained at a single site (around the Old Cut field station; Figure 1). Daily locations were obtained on the mainland and inhabited areas at the base of the peninsula, by conducting daily searches for individuals along roads and trails. Individuals located manually were ‘localized’ to within a small area (<2 ha) through triangulation or (more rarely) direct observation. For the peninsula itself we used an all-terrain vehicle to drive along the beach, searching for individuals on foot from high dunes every 1–2 km, every day or second day when weather did not permit searching.

The 5-element antenna (AF Antronics Inc., Urbana, Illinois: H-plane 61°, isotropic gain 10.1 dBi, front-to-back ratio >20 dB) had broader, shorter sensitivity whereas the 9-element (Lindsay Antennas, Lindsay, Ontario: H-plane 49°, isotropic gain 12.5 dBi, front-to-back ratio 20 dB) had narrower, longer sensitivity. Antennae were located at least 10 m above ground on all towers. Recorded data comprised a tag number, date, location, antenna number, and signal strength (an index from ∼20 – 255 linearly related to the log of the received signal in dB) and time, which was coordinated between receivers by internal GPS clocks.

### Calibration tests

Calibrations were conducted on calm days by attaching a working transmitter to a partially frozen specimen and raising it in a large helium-filled balloon tethered behind a boat. Transects were run along the cardinal directions at 1 km intervals away from a stationary tower until the signal was no longer detected. Every 1 km the specimen was raised to varying altitudes from 50 to 200 m. The tag signal was simultaneously recorded by the automated tower and a portable receiver in the boat which was coordinated by an internal GPS and clock. These tests suggested a maximum detection distance (under ideal conditions, when targets were in the air and in line of sight of the towers) of ∼12 km. Multiple simultaneous detections from flights of certain individuals between towers ∼30 km apart suggest that this detection distance was sometimes exceeded in our data.

### Animals and transmitters

We deployed 322 transmitters on seven species (Table 1). Individuals were captured using mist nets (32 mm) at three locations (Figure 1). Animals were tagged during the peak of their individual migrations; 25 September– 9 November (fall 2008; previously reported in [6]), 15 April – 7 June (spring 2009), and 20 August – 15 October (fall 2009). We used three transmitter types (always <4% body weight) from Lotek: ANTC-M1-1 and ANTC-M2-1 (fall 2008, spring 2009; battery life 18 d, weight 0.75 g, burst rates 4.5 – 5.5 s), and NTQB-1 (fall 2009; battery life 28 d, weight 0.29 g, burst rates ∼7 s).

**Table 1:** Flight and movement data from 322 individuals of 6 species of birds and 1 species of bat tracked during fall 2008, spring 2009, and fall 2009 using radio-telemetry at Long Point, Ontario, Canada.

species	season	N	probable departures	stopover flights			other movements	
			n	n (%N)	observed after 24 h	mean (max) distance - km	n	mean (max) distance - km
Swainson's Thrush *Catharus ustulatus*	Fall 2008	30	21	2 (7)	1	10.0 (15)	1	2.0
	Spring 2009	50	36	4 (8)	3	2.4 (5)	0	-
Hermit Thrush *Cathrarus guttatus*	Fall 2008	39	27	8 (21)	5	7.3 (29)	1	2.0
	Spring 2009	31	22	5 (16)	0	10.3 (26)	0	-
Black-throated Blue Warbler *Dendroica caerulescens*	Fall 2009	83	49	46 (47)	23	6.1 (30)	6	1.7 (2.5)
White-throated Sparrow *Zonotrichia albicollis*	Spring 2009	19	9	1 (5)	0	21.0	1	2.3
White-crowned Sparrow *Z. leucophrys leucophrys*	Spring 2009	16	14	0 (0)	0	-	4	1.6 (2.4)
Northern Saw-whet Owl *Aegolius acadicus*	Fall 2008	24	12	24 (46)	17	7.1 (19)	-	-
Silver-haired Bat *Lasionycteris noctivagans*	Fall 2009	30	23	7 (23)	1	6.7 (18)	-	-

We show the number of individuals tagged (N) and the number of individuals that were eventually detected departing (probable departures, n). For stopover flights, we report the number flights observed (and the percentage of N individuals making those flights) and the number of flights that occurred 24 h or more after tagging. For both stopover flights and other movements we summarize the distances moved.

We used loop harnesses secured around the legs to attach transmitters to the passerines [25] and loosely around the wings to attach transmitters to the owls [26]. For bats, we affixed the transmitter to the upper back using ostomy bonding cement (Torbot; Cranston, Rhode Island) after removing some fur [27].

Similar proportions of adults and young were tagged for thrushes and the warblers. For sparrows, we targeted young (second-year) individuals; by chance, most owls were adult (22/24), and most bats were young (28/30). For birds, we attempted to balance the numbers of fat and lean animals among sites. No attempt was made to balance samples by sex, except for the warbler and bat species where sex could be unambiguously determined.

### Analysis of telemetry data

**Figure 2:**
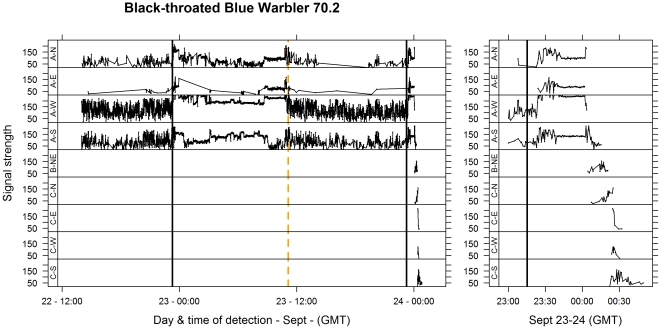
A probable migratory departure for a Black-throated Blue Warbler. Variation in signal strength (arbitrary scale from 0–255) by multiple antennae on three towers (see Figure 1 for locations) is plotted by time. Black vertical lines are sunset; orange dashed lines are sunrise. Left most panel shows all detections in ∼48 h prior to departure; the right panel repeats the last ∼70 minutes of detection (during departure) on an expanded time scale. Reading from left to right (left hand panel), the individual is active up to sunset (high variation in signal strength) and relatively inactive during the night (low variation in signal strength). At sunset on 24 Sept (right hand panel) there is a rapid increase followed by a short period (∼30 min) of low variation in signal strength suggesting the individual moved to a more exposed location just prior to initiating a migratory flight of ∼35 min. The migratory flight is shown by a sharp increase in signal strength at ∼0015 (see peaks on A–S and A–N antennas), followed by detections on tower B and C situated to the South. Final detections show declining signal strength on the C–S antenna suggesting that the individual flew directly south across Lake Erie.

Digital encoding of tag numbers allowed simultaneous monitoring of up to ∼25 individuals within a small area. Individuals within range of the receiver were monitored continuously. When individuals were on or near the ground, they could be detected within a maximum of 0.5–2 km depending on their location relative to the receiving antennae. To identify flights, we visually inspected plots of signal strength versus time for each individual. Flights were defined by a sharp increase in signal strength at one or more antennae, followed by a change in the detection pattern from the antennae on one or more towers (Figure 2). When an individual was detected over several minutes, the sequence of detections revealed the approximate direction and distance of a flight.

Binary data files (precise to fractional seconds for SRX 600 units) were downloaded to a computer and examined daily for movements or departures assisting in manual searches. To ensure that our data only contained valid detections, we post-processed detections by determining the precise interval between transmissions on each tag (the burst rate) and then removed signals not matching that interval to within ∼0.02 s. We carefully inspected plots (described above) of all individuals to ensure that we did not eliminate apparently valid detections that indicated movement.

### Ethics Statement

The methods used in this study adhere to the Ornithological Council's guidelines to the use of wild birds in research and the Canadian Council on Animal Care, and were approved by Animal Care Committees at Acadia University (13-04A#2R#3) and the University of Western Ontario (#2008-003; #2006-14). Other permits were from Environment Canada (Canadian Wildlife Service #10169) and the Ontario Ministry of Natural Resources (#1050823).

## Results

**Figure 3:**
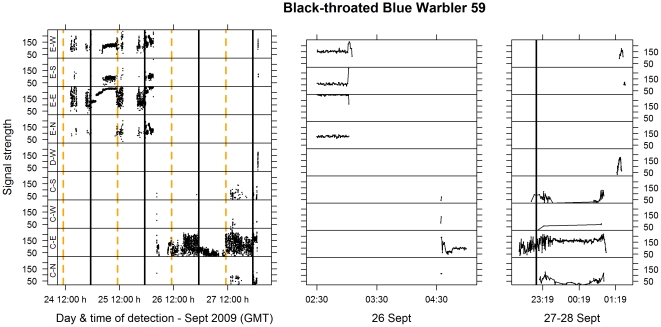
A stopover flight and probable migratory departure for a Black-throated Blue Warbler. Figure notation as for Figure 2. The individual was tagged at the Tip (near tower E) on the morning of 24 Sept and remained in the vicinity of the Tip for two days. During the night, signal strength is generally constant, whereas during the day, signal strength varies continuously as the individual moves around. Approximately mid-way through the night of 26 Sept the individual makes a stopover flight – It flies west and settles near tower C. It remains there for two more full days, before departure after sunset on 28 Sept. The middle and right hand panels provide a more detailed view of the detections for the stopover flight and departures, respectively. Note that the stopover flight (middle panel) was ∼30 km over 1.5 h (flight speed of 20 km/h) suggesting that the movement was a single flight. The individual was relocated after the movement. Note that for the departure flight (right panel) the individual was detected leaving the vicinity of tower C to the east (last detections on antenna C–E), then traversed the beam of tower D–W while simultaneously being detected on the west antenna at the Tip (E–W). It was last detected traversing the beam of the south antenna at the Tip (E–S). The pattern suggests a SE departure over Lake Erie.

Flights were classified into three types. ‘Stopover flights’ were sustained (>2 min) flights in any direction that were followed by an automated or manual re-detection within the extent of the monitored area (the stopover landscape) and at least 1 km from their last location (Figure 3). (Stopover flights comprise what we termed ‘local’ movements and ‘landscape-scale movements’ in previous work [6], but in this paper we do not distinguish between these two types of flights based on distance). ‘Probable migratory departures’ (‘true departures’ in previous work [6]) were sustained nocturnal flights in a seasonally appropriate direction (i.e. with a North component in spring or a South component in fall) with no subsequent observations (Figure 2, 3). Sustained nocturnal flights in seasonally inappropriate directions were not included in this group as they could have been ‘reverse migrations’ [21], [22], and therefore not a direct continuation of migration toward the wintering grounds. ‘Ambiguous flights’ were those where individuals were not observed again, but where there were too few detections to clearly establish a direction, or where detection suggested movement in a seasonally inappropriate direction. Ambiguous flights therefore include some combination of stopover flights, probable migratory departures, and reverse migrations and, in this study, are not subject to further analysis. Ambiguous flights were more frequent in data sets collected in fall 2008 and spring 2009, when we did not have towers on the adjacent mainland (and so were less able to capture landscape-scale relocations when they occurred). Eliminating such flights from the data set means that our estimate of the frequency of landscape-scale movements is conservative, in that, because of the locations of the towers, it is likely that true migratory departures had a higher probability of being detected than landscape-scale movements.

Individuals also moved (via short flights during the day) beyond 1 km from their original capture site. These movements were detected when individuals changed locations over subsequent days, but where sustained flights were not observed on the digital array. We collectively termed these ‘other movements’.

Across all species, we detected 411 flights, 101 of which were classified as ambiguous. The remaining 310 flights (by 245 individuals) comprised 213 probable migratory departures and 97 stopover flights (Table 1).

Stopover flights were observed in both spring and fall, and for all species except White-crowned Sparrow. Among the other species, between 5% and half of all individuals were observed making stopover flights (Table 1); 15 individuals (8 owls and 7 warblers) were observed making more than one such flight. Twenty-two individuals (7%) relocated >10 km from the point of initial capture and 76 (24%) relocated at least 1 km distant (Table 1). Individuals making stopover flights moved from each of the three capture sites and both toward and away from the expected seasonal heading. In addition, 13 individuals (4%) made ‘other movements’ resulting in displacements as much as 2.5 km from their point of initial capture (Table 1).

About half of stopover flights (52%) occurred 24 h or more after first capture (Table 1) and most were at night. Thirteen of 66 (20%) passerine stopover flights occurred at or near dawn. We do not attempt to compare flights among species because the extent and setup of the digital array varied considerably between the three seasons, and because the particular geography of Long Point (a peninsula on the north side of a large lake) means that with our arrangement of tower locations and antenna directions, stopover flights were more easily detected in fall than in spring. Further, we do not attempt here to fully deal with the many various extraneous factors influencing behaviours of individuals; these are the subject of more in-depth analyses in separate papers (e.g. [6]).

## Discussion

Our results reveal that during a stopover bout, migrants from multiple taxa with diverse migration strategies frequently undertake flights from monitored stopover sites that do not result in a continuation of migration, but rather are movements between stopover sites within a broader stopover landscape. Similar results have been observed for shorebirds where individuals regularly undertake ‘within-stopover’ movements [8], [28] but the behaviour is not usually reported for passerines and even more rarely (if at all) for owls and bats. The implication of this result is that to properly quantify migratory behaviour at stopover, researchers need to ensure that they are surveying at spatial scales that fully represent the stopover landscape.

Several previous authors have emphasized the importance of scale in understanding stopover ecology [28]–[30], but quantitative measures of the spatial scale of stopover bouts are still rare. We suggest that much current knowledge of stopover rests on two implicit, but unreliable assumptions: 1) that fine-scale monitoring of migrants at an individual stopover site (usually ∼1 km^2^ or the size of a mist-netting or telemetry search area) provides representative information about their behaviour and ecology throughout a complete stopover bout within a broader landscape, and 2) that nocturnal flights away from monitored sites represent a continuation of migration. Violation of either of these assumptions changes both the interpretation of stopover behaviours as well as views of how stopover landscapes should be studied and protected. For example, if the scale of stopover is broader than measured, then estimates of stopover duration will be biased low. Furthermore, if some, but not all individuals re-locate to other parts of the landscape during a stopover bout, then the assumed importance of landscape components where individuals are typically first encountered, such as lakeshores, will also be incorrectly valued. Our results demonstrate that non-migratory nocturnal flights from monitored sites do occur, and that such flights can result in the spatial scale of stopover extending tens of kilometres or possibly more, in any direction from the site of first capture.

One purpose of stopover flights may be to enable individuals to relocate to more favorable habitat [29], [31]. Habitat choice has been proposed as a mechanism to explain reverse nocturnal migrations [20], [32] and diurnal morning flights [18] that are often observed within 24 h of initial capture. Given that over half of our observed stopover flights occurred >24 h after arrival, it is possible that assessment of habitat and subsequent relocations continue later into the stopover period than has previously been suggested. Individuals undertaking these flight may be making more prolonged habitat assessments [24], and(or) habitat conditions or needs may change with time [17]. Another purpose of stopover flights may be pre-departure evaluation of weather conditions aloft [33], [36]. Such nocturnal exploratory flights may result in subsequent relocation if a migratory flight does not occur. Finally, the specific behaviours will almost certainly differ among individuals, species, seasons, locations and even depend on the stage of the migratory journey. While some of the proximate mechanisms for these behaviours have been studied for some species [34], [36] they are not always considered in the context of influencing re-location within stopover.

The concept of a ‘stopover bout’ is poorly defined in the literature, and is partly a function of how broadly or narrowly one defines a ‘migratory flight’. For example, if any type of nocturnal flight to a new location is considered to be a ‘migratory flight’, then the time spent at the new location, even if only 1–2 km distant, represents a new stopover bout. The handful of studies that have directly measured migratory flights indicate that the average distance of a single migratory flight for a nocturnal migrant ranges from ∼170 km [1], [37], [38] to 270 km [39]. However, it remains to be seen whether the shorter flights that we have observed here are the lower end of a continuum of distances flown, or whether they represent a distinct behavioural phenomenon. We do have some evidence that, for the two *Catharus* thrushes [6], ‘stopover flights’ are distinguishable from true migratory flights, in that they occur at any time of the night and in multiple directions, as opposed to shortly after civil twilight and in a seasonally appropriate direction.

Finally, it may be argued that ambiguous flights in seasonally inappropriate directions are simply ‘reverse migrations’, but with our data, it is impossible to differentiate these from relocations beyond the extent of our study area (e.g. relocations beyond about 30 km). We would argue however that some observed ‘reverse migrations’ may simply be relocations within the broader stopover landscape, and that further study is required to identify the underlying behavioural motivation of these various movements.

To clarify these behaviours and better classify the movements, we propose that studies of nocturnal migrants define two types of stopover: ‘true stopover’ being the time and space occupied by an individual between true migratory flights (acknowledging that the definition of a migratory flight may be argued), and ‘apparent stopover’ being the time and space occupied by an individual between any nocturnal flights. True stopover thus comprises a complete stopover bout throughout a stopover landscape, whereas apparent stopover may only refer to an individual's presence at any given stopover site within that landscape. Regardless of the actual definition of stopover, the fact that some individuals relocate within a broad landscape during migration is of relevance to the ecology and conservation of migrant species.

The taxonomic and seasonal ubiquity of within-stopover movements that we observed has important implications for migration theory, conservation planning, and research design, especially given the significance of stopovers for conservation [3], [35]. Properly considering the scale of stopover and the function of different types of nocturnal flights will contribute to better empirical research and improved plans for protection of critical migratory habitats.

## References

[1] Wikelski M, Tarlow EM, Raim A, Diehl RH, Larkin RP (2003). Avian metabolism: costs of migration in free-flying songbirds.. Nature.

[2] Newton I (2006). Can conditions experienced during migration limit the population levels of birds?. Journal of Ornithology,.

[3] Faaborg J, Holmes RT, Anders AD, Bildstein K, Dugger KM (2010a). Conserving migratory land birds in the New World: Do we know enough?. Ecol App.

[4] Faaborg J, Holmes RT, Anders AD, Bildstein K, Dugger KM (2010b). Recent advances in understanding migration systems of New World land birds?. Ecol Mono.

[5] Holberton RL, Dufty AM (2005). Hormones and Variation in Life History Strategies of Migratory and Non-Migratory Birds..

[6] Mills AM, Thurber BG, Mackenzie SA, Taylor PD (2011). Passerines use nocturnal flights for landscape-scale movements during migration stopover.. Condor.

[7] Butler RW, Shepherd PC, Lemon MJF (2002). Site fidelity and local movements of migrating Western Sandpipers on the Fraser River Estuary.. Wils J Orn.

[8] Sprague AJ, Hamilton DJ, Diamond AW (2008). Site safety and food affect movements of Semipalmated Sandpipers (*Calidris pusilla*) migrating through the upper Bay of Fundy.. Avian Conservation and Ecology.

[9] Moore FR, Aborn DA (1996). Time of departure by Summer Tanagers (*Piranga rubra*) from a stopover site following spring trans-gulf migration.. Auk.

[10] Bächler E, Schaub M (2007). The effects of permanent local emigration and encounter technique on stopover duration estimates as revealed by telemetry and mark-recapture.. Condor.

[11] Goymann W, Spina F, Ferri A, Fusani L (2010). Body fat influences departure from stopover sites in migratory birds: evidence from whole-island telemetry.. Biol Lett.

[12] Åkesson S, Alerstam T, Hedenström A (1996). Flight initiation of nocturnal passerine migrants in relation to celestial orientation conditions at twilight.. J Avian Biol.

[13] Chernetsov N, Mukhin A (2006). Spatial behavior of European robins during migratory stopovers: a telemetry study.. Wils J Orn.

[14] Matthews SN, Rodewald PG (2010). Urban forest patches and stopover duration of migratory Swainson's thrushes.. Condor.

[15] Schaub M, Jenni L, Bairlein F (2008). Fuel stores, fuel accumulation, and the decision to depart from a migration stopover site.. Behav Ecol.

[16] Ktitorov P, Tsvey A, Mukhin A (2010). The good and the bad stopover: behaviours of migrant reed warblers at two contrasting sites.. Behav Ecol and Soc.

[17] Paxton KL, Van Riper C, O'Brien C (2008). Movement patterns and stopover ecology of Wilson's Warblers during spring migration on the lower Colorado River in southwestern Arizona.. Condor.

[18] Wiedner DS, Kerlinger P, Sibley DA, Holt P, Hough J (1992). Visible morning flights of Neotropical landbird migrants at Cape May, New Jersey.. Auk.

[19] Alerstam T (1978). Reoriented bird migration in coastal areas: dispersal to suitable resting grounds?. Oikos.

[20] Richardson W (1978). Timing and amount of bird migration in relation to weather: a review.. Oikos.

[21] Åkesson S (1999). Do passerine migrants captured at an inlands site perform temporary reverse migration in autumn?. Ardea.

[22] Zehnder S, Åkesson S, Liechti F, Bruderer B (2002). Observation of free-flying nocturnal migrants at Falsterbo: occurrence of reverse flight directions in autumn.. Avian Science.

[23] Chernetsov N, Titov N (2000). Design of a trapping station for studying migratory stopovers by capture-mark- recapture analysis.. Avian Ecol Behav.

[24] Tsvey A, Bulyuk VN, Kosarev (2007). Influence of body condition and weather on departures of first-year European robins, *Erithacus rubecula*, from an autumn migratory stopover site.. Behav Ecol Socio.

[25] Fair J, Paul E, Jones J, Eds (2010). Guidelines to the Use of Wild Birds in Research..

[26] Rappole JH, Tipton AR (1991). New harness design for attachment of radio transmitters to small passerines.. J Field Orn.

[27] Amelon SK, Dalton DC, Millspaugh JJ, Wolf SA, Kunz TH, Parsons S (2009). Radiotelemetry: Techniques and Analysis.. Ecological and Behavioral Methods for the Study of Bats.

[28] Farmer AH, Parent AH (1997). Effects of the landscape on shorebird movements at spring migration stopovers.. Condor.

[29] Moore FR, Gauthreaux SA, Kerlinger P, Simons TR, Martin TE, Finch DE (1995). Habitat requirements during migration: important link in conservation.. Ecology and Management of Neotropical Migratory Birds.

[30] Buler JJ, Moore FR, Woltmann S (2007). A multi-scale examination of stopover habitat use by birds.. Ecology.

[31] Chernetsov N (2006). Habitat selection by nocturnal passerine migrants en route: mechanisms and results.. J Ornithol.

[32] Åkesson S, Karlsson L, Walinder G, Alerstam T (1996). Bimodal orientation and the occurrence of temporary reverse bird migration during autumn in south Scandinavia.. Behav Ecol Socio.

[33] Richardson WJ, Gwinner E (1990). Timing of bird migration in relation to weather: updated review.. Bird Migration: Physiology and Ecophysiology Berlin: Springer-Verlag.

[34] Sandberg R, Moore FR (1996). Migratory orientation of red-eyed vireos, *Vireo olivaceus*, in relation to energetic condition and ecological context.. Behav Ecol Sociobiol.

[35] Hutto RL (2000). On the importance of en-route periods to the conservation of migratory landbirds.. Stud Avian Biol.

[36] Schmaljohann H, Becker JJP, Karaardic H, Liechti F, Naef-Daenzer B (2010). Nocturnal exploratory flights, departure time, and direction in a migratory songbird.. Journal of Ornithology.

[37] Ellegren H (1993). Speed of migration and migratory flight lengths of passerine birds ringed during Autumn migration in Sweden.. Ornis Scandinavia.

[38] Cochran WW, Wikelski M, Greenberg R, Marra P (2005). Individual migratory tactics of New World Catharus thrushes: current knowledge and future tracking options from space.. Birds of Two Worlds: The ecology and evolution of migration.

[39] Stutchbury BJM, Tarof SA, Done T, Gow E, Kramer PM (2009). Tracking long-distance songbird migration by using geolocators.. Science.

